# Characterisation of Castration-Resistant Cell-Derived Exosomes and Their Effect on the Metastatic Phenotype

**DOI:** 10.3390/cancers17010141

**Published:** 2025-01-04

**Authors:** Jorge Recio-Aldavero, Lorena Parra-Gutiérrez, Laura Muñoz-Moreno, Irene D. Román, María Isabel Arenas, Ana M. Bajo

**Affiliations:** 1 Unidad de Bioquímica y Biología Molecular, Departamento de Biología de Sistemas, Campus Científico-Tecnológico, Universidad de Alcalá, 28805 Alcalá de Henares, Spain; 2 Unidad de Biología Celular, Departamento de Biomedicina y Biotecnología, Campus Científico-Tecnológico, Universidad de Alcalá, 28805 Alcalá de Henares, Spain

**Keywords:** prostate cancer, exosomes, Raman-SERS, gamma-glutamyl transpeptidase, matrix metalloproteinase

## Abstract

Background/Objectives: Prostate cancer (PCa) is characterised by its progression to a metastatic and castration-resistant phase. Prostate tumour cells release small extracellular vesicles or exosomes which are taken up by target cells and can potentially facilitate tumour growth and metastasis. The present work studies the effect of exosomes from cell lines that are representative of the different stages of the disease on the tumoral phenotype of PC3 cells. Methods: Exosomes were isolated by ultracentrifugation from human prostate epithelial cells (RWPE-1) and androgen-dependent PCa cells (LNCaP) and castration-resistant PCa cells (CRPC) with moderate (DU145) or high (PC3) metastatic capacity. The biophysical and biochemical properties of the exosomes were characterised as well as their effects on PC3 cell viability and migration. Results: The study of the exosomes of prostate cell lines shows heterogeneity in their size, presenting in some of them two types of populations; in both populations, a larger size in those derived from PC3 cells and a smaller size in those derived from non-tumourigenic prostate cells were detected. Differences were found in the physical properties of those derived from healthy and PCa cells, as well as between cells representative of the most aggressive stages of the disease. The highest gamma-glutamyl transferase (GGT) activity was observed in androgen-dependent cells and differences in the pro-metalloproteinases (MMP) activity were detected in healthy cells and in castration-resistant cells with moderate metastatic capacity with respect to PC3 cells. The treatment of PC3 cells with their own exosomes increased PC3 cell viability and migration. Conclusion: Exosomes represent a promising field of research in the diagnosis, prognosis, and treatment of prostate cancer.

## Simple Summary

Prostate cancer (PCa) ranks as the second most common and fifth most deadly cancer among men globally. A defining feature of this cancer is its progression to a castration-resistant and metastatic state. The prostate-specific antigen (PSA), the primary biomarker for PCa diagnosis and screening, lacks specificity. Therefore, there is a need for further knowledge of new and more specific biomarkers, including exosomes. The aim of our study was to perform biochemical and biophysical characterisation of exosomes derived from cells representative of prostate cancer progression. In addition, we also wanted to study the role of these extracellular vesicles in the metastatic phenotype of castration-resistant cells. The results obtained suggest that the exosomes may be biomarkers for the diagnosis and prognosis of PCa.

## 1. Introduction

Prostate cancer (PCa) ranks as the second most common and fifth most deadly cancer among men globally. The Global Cancer Observatory (GCO, formerly GLOBOCAN) estimates indicate that its prevalence and mortality rates are expected to rise in the coming decades. A defining feature of this cancer is its progression to a castration-resistant and metastatic state [1]. Additionally, the prostate-specific antigen (PSA), the primary biomarker for PCa diagnosis and screening, lacks specificity [2]. As a result, significant progress has been made in developing modified PSA tests such as PSA velocity, PSA density, 4Kscore, PSA glycoprofiling, the Prostate Health Index, and the STHLM3 test [3]. Similarly, it has been stated that the traditional PSA cut-off point of 3 ng/mL should be abandoned in favour of age-specific reference ranges [4]. This limited understanding poses challenges for identifying therapeutic targets and developing effective drugs.

New molecular biomarkers of prostate cancer are beginning to play an important role in improving diagnosis and treatment [5], and among these, exosomes emerge as one of the most promising. Different subtypes of extracellular vesicles have been described according to their size: small extracellular vesicles (S-EV) or exosomes below 200 nm, and large extracellular vesicles (L-EV) or oncosomes above 200 nm [6]. Exosomes facilitate intercellular communication by fusing with the membranes of recipient cells and contain a variety of biological molecules, including proteins, lipids, and nucleic acids. Tetraspanins, including CD9 and CD63 as well as lysosome-associated membrane protein (LAMP-2), are used as exosomal markers. These vesicles are secreted by most cell types and have garnered significant attention in biomarker discovery due to their detectability and remarkable stability in various body fluids—blood, urine, semen, and saliva—and in cell culture medium [7,8].

After release, exosomes are taken up by neighbouring or distant cells. The miRNAs contained within these exosomes modulate various processes, such as interfering with tumour immunity and altering the microenvironment, which can potentially facilitate tumour growth, invasion, metastasis, angiogenesis, and drug resistance. Consequently, exosomes play an important role in the regulation of cancer progression through the transfer of miRNAs [9]. In the context of prostate cancer, prostate-derived exosomes serve as a rich source of molecular markers—proteins, RNA, and miRNA—making them valuable for both diagnosis and prognosis of the disease [10].

There are some proteins that serve various roles in the pathology, diagnosis, and treatment of prostate cancer, making them critical subjects of research and clinical interest. Among them, we can highlight prostate-specific membrane antigen (PSMA), matrix metalloproteinases (MMPs), gamma-glutamyl transferase (GGT), p53, and N-cadherin. PSMA is a transmembrane protein that is anchored in the cell membrane of prostate cancer epithelial cells and whose expression increases in parallel with prostate cancer progression [11]. MMPs are zinc and calcium-dependent endopeptidases capable of degrading several components of the extracellular matrix, such as collagen, fibronectin, and laminin; increased activity of MMP-2 and MMP-9 has been shown to promote cell migration and invasion in PCa [12]. GGT is a membrane protein with enzymatic activity that contributes to the metabolism of glutathione (GSH), which plays a critical physiological role in protecting cells against oxidative stress. GGT is primarily found on the surface of cancer cells such as prostate cancer, without being overexpressed in normal cells or tissues [13]. In addition, serum GGT is found to be significantly and independently associated with shorter overall survival in patients with metastatic prostate cancer [14]. N-cadherin is a hallmark of epithelial-to-mesenchymal transition, resulting in the acquisition of an aggressive tumour phenotype [15]; N-cadherin has been reported to be upregulated and associated with metastasis and poor prognosis in prostate cancer patients [16].

It has been shown that prostate tumour cells release exosomes with different contents compared to those from healthy cells. When these exosomes are taken up by target cells, they can induce various changes, such as promoting cell differentiation and growth, altering the immune response within tumours, and enhancing the migratory and invasive capabilities of cancer cells [17]. The aim of the study was to characterise, physically and biochemically, exosomes derived from cells representative of different stages of prostate cancer. For this purpose, exosomes were isolated from cells representing (i) human prostate epithelial cells (RWPE-1), and (ii) androgen-dependent PCa cells (LNCaP) and castration-resistant PCa cells (CRPC) with moderate (DU145) or high (PC3) metastatic capacity. The biophysical properties (DLS, ELS, and Raman-SERS) and biochemical characteristics (MMPs and GGT) of exosomes from different cell lines were studied. The role of exosomes derived from castration-resistant cells in cell viability and migration of PC3 cells has also been evaluated. In addition, N-cadherin expression has been determined in exosomes from both healthy and castration-resistant PCa cells.

## 2. Materials and Methods

### 2.1. Cell Culture

Four human prostate cell lines were used. Cell lines were obtained from the American Type Culture Collection. Non-neoplastic, immortalised adult human prostatic epithelial cells, RWPE-1 (passages 5–15, ATCC CRL-11609) are androgen-responsive and show many characteristics of nontumor cells. The three human PCa cell lines used exhibit different features of PCa progression. An androgen-responsive cancer cell line, LNCaP (passages 15–20, ATCC CRL-1740) and two androgen-unresponsive cell lines, DU145 (passages 8–14, ATCC HTB-81), derived from brain metastasis and with moderate metastatic potential, and PC3 (passages 9–25, ATCC CRL-1435), derived from bone metastasis with high metastatic potential. RWPE-1 cells were maintained in complete keratinocyte serum-free medium containing 50 μg/mL bovine pituitary extract and 5 ng/mL human epidermal growth factor (EGF). The rest of the cell lines were cultured in RPMI-1640 medium supplemented with 10% FBS. All culture media contained 1% penicillin/streptomycin/amphotericin B (Life Technologies, Alcobendas, Madrid, Spain). Culture was carried out in a humidified 5% CO_2_ environment at 37 °C. To produce the exosomes of the different cell lines and isolate them, the cell lines had to be cultured in the corresponding media supplemented with exosome-free medium and 1% antibiotic/antimycotic (penicillin/streptomycin/amphotericin B) (Life Technologies). After 72 h, the culture medium was collected and stored at −80 °C until use.

### 2.2. Isolation of Media-Derived Exosomes

To isolate the exosomes from the cell culture media, an initial centrifugation was performed at 100× *g* for 10 min to remove cell debris and apoptotic bodies. The supernatant was then passed through a 0.20–0.22 μm filter to remove larger particles and, finally, ultracentrifugation was performed at 140,000× *g* for 2 h at 4 °C to recover the sediment with the exosomes. Subsequently, the exosomes were resuspended in 200 μL of sterile PBS and stored at −80 °C. The total protein concentration of the exosome samples was determined by bicinchoninic acid (BCA) assay following the protocol provided by the BCA Protein Assay Kit (Thermo Scientific TM, Alcobendas, Madrid, Spain).

### 2.3. Transmission Electron Microscopy (TEM)

Following isolation, exosomes were prepared for visualisation using transmission electron microscopy (TEM). First, they were dissolved in phosphate-buffered saline (PBS) and adsorbed onto carbon-formvar-coated nickel grids. To preserve their morphology and allow for further immunocytochemical analysis, the grids were fixed with a solution containing 0.5% glutaraldehyde and 2% paraformaldehyde. After washing, non-specific antibody binding was reduced by blocking the grids with 3% normal donkey serum (NDS). Subsequently, the exosomes were incubated overnight with a primary antibody specific to the proteins CD9 and CD63 (diluted 1:50, Santa Cruz). Following another PBS wash, the sections were incubated with a secondary antibody 10 nm gold-labelled goat anti-mouse IgG (diluted 1:100, Biocell) for 1 h at room temperature. Finally, the grids were counterstained with a 1% saturated solution of uranyl acetate, and the exosomes were visualised under a Zeiss EM-10 TEM.

### 2.4. Western Blotting

Exosomal proteins isolated from cell culture media were analysed using Western blotting. First, electrophoresis gels were prepared: stacking gel (5%) and resolving gel (12%) of acrylamide-bisacrylamide. Samples were prepared by mixing 100 μg of protein with loading buffer (50% glycerol, 15% SDS, 25% β-mercaptoethanol, 50 mM Tris-HCl (pH 6.8), and 0.0125% bromophenol blue) and denatured at 95 °C for 5 min. Proteins were then separated by electrophoresis under denaturing conditions (SDS-PAGE) and transferred to PVDF membranes in Trans-Blot^®^ TurboTM for 30 min at 25 V. The membranes were incubated first with blocking solution (5% skimmed milk in PBS) for 1 h under agitation and then with the primary antibodies at different dilutions (Table 1) at 4 °C overnight. The next day, the membranes were washed with PBS, incubated with the corresponding secondary antibody at 1:5000 dilution for 1 h at room temperature, washed again with PBS, and finally incubated for 2 min with ECL reagent prepared with the Elabscience Excellent Chemiluminescent Substrate kit. The membranes were visualised on Bio-Rad’s ChemiDoc imaging system and analysed using Image Lab 6.1 software.

### 2.5. Dynamic Light Scattering (DLS) and Electrophoretic Light Scattering (ELS)

The instrument used to perform both techniques was the Zetasizer Nano ZS (Malvern UK). The DLS assay was used to study the size distribution of the isolated exosomes. The ELS assay was used to assess the zeta potential (a measure of the effective electric charge on the surface of vesicles) of previously isolated exosomes. To measure the size and its distribution, after stabilisation of the instrument with respect to the laser and temperature, 90 µL of the exosome sample (previously diluted 10-fold) was loaded into a ZEN0040 cuvette and introduced into the Zetasizer Nano ZS instrument. To ensure reproducibility and standardisation, the following parameters were employed: laser wavelength set at 633 nm, temperature control activated, peak radius range defined by a low cutoff of 0.4 nm and a high cutoff of 10,000 nm, auto-attenuation time limit set to 10 s, polydispersity calculation enabled, temperature set to 15 °C, and a waiting time of 2 min. The DLS measurement itself was comprised of 3 acquisitions, each lasting 40 s.

The conditions used to measure the zeta potential were to load a volume of 900 μL of sample into a DTS1070 cuvette and to select a temperature of 15 °C with a stabilisation time of 120 s. Three ELS measurements were performed in automatic mode.

### 2.6. Surface-Enhanced Raman Spectroscopy (SERS)

Since the Raman technique has low sensitivity in liquid samples, it has been improved by amplifying the signal using the surface-enhanced Raman spectroscopy (SERS) analytical technique. Silver nanoparticles (AgNP) were synthesised by chemical reduction of an AgNO_3_ solution (50 mL, 10^−3^ M) with a 1% trisodium citrate solution (1 mL). The reaction was carried out in a round bottom flask at reflux, at a constant temperature of 250 °C, for 1 h, until the reaction solution turned a grey-orange metallic colour. Finally, to concentrate the silver nanoparticles formed, 1.5 mL of the solution was centrifuged at 4020× *g* for 8 min at 4 °C. Finally, 100 µL was collected from the bottom of the tube containing the concentrated AgNPs. The AgNPs generated were analysed using the dynamic light scattering (DLS) and the transmission electron microscopy (TEM) technique to measure the size of the AgNPs. The measures were performed on a Whatman paper support: 5 µL of AgNP and 5 µL of sample were added. The internal content of the exosomes was also analysed using the Raman-SERS technique. For this, the exosome membrane was broken by means of a lysis buffer (1% Triton X-100), which was added to the sample and kept overnight to guarantee its effect.

The equipment used was a B&WTek GlacierX CCD Raman spectrometer with 2048 pixels, cooled by a B&WTek Peltier cell, coupled with optical fibre to a microscope with an epi-illumination system and B&WTek Cleanlaze 785 nm excitation. Measurements were performed in dark conditions for 40 s, with a 20× epifluorescence objective and at a power of 20 mW. The spectra were obtained with BWSpec 4 software in .txt and .spc format (*y*-axis, “Dark Subtracted”; *x*-axis, “Raman Shift 8-3170”), visualised, and the characteristic peak areas were calculated with SpectraGryph 1.2.

Raman spectra of the exosome samples were analysed by Principal Component Analysis (PCA) and PCA-Linear Discriminant Statistical Analysis coupled to PCA (PCA-LDA) with the Unscrambler X 10.4 software. PCA analysis is an unsupervised learning algorithm designed to identify the components that maximise variance within the data (i.e., variance within variables) in order to highlight differences. Unlike PCA, LDA is used to find the feature subspace (i.e., a linear combination of the observed variables) that maximises the separation between classes by optimising between-class variance. Linear Discriminant Analysis (LDA) coupled with Principal Component Analysis (PCA) allows us to improve the classification of complex data into clusters. Once it is determined to which of the established categories each sample belongs, we can obtain precise information on the specificity and sensitivity of the separation by components, which confirms the accuracy of the model in the separation of samples.

For this purpose, a chemometric analysis was performed to compare groups of exosome samples according to their principal components (PCA) [18]. As the PCA is an unsupervised analytical method (there is no previous information on how to classify the groups of samples) we need to accompany it with an LDA in which we will classify the group to which each sample belongs [19].

### 2.7. Gamma-Glutamyl Transpeptidase (GGT) Activity

The GGT activity of the serum and serum-derived exosomes was determined with the Spinreact Quantitative Determination of GGT kit according to the manufacturer’s instructions. Briefly, GGT catalyses the transfer of the γ-glutamyl group from γ-glutamyl-p-nitroanilide to the acceptor glycylglycine, according to the following reaction:

γ-L-Glutamyl-3-carboxy-4-nitroanilide+Glycylglycine→γ-L-Glutamyl-glycylglycine+2-Nitro-5-aminobenzoic acid

The rate of formation of 2-nitro-5-aminobenzoic acid, measured photometrically, is proportional to the catalytic concentration of GGT present in the sample [20]. All GGT activity measurements were performed with 40 μg of protein per sample.

### 2.8. Zymography Assay

The gelatinase activity of the media collected after the treatments was evaluated by Zymography. For this, 50 μg of protein mixed with loading buffer composed of 50 mM Tris-HCl (pH 6.8), 10% glycerol, 0.01% SDS and 0.01% bromophenol blue was loaded onto a 0.1% gelatine copolymerised acrylamide/bisacrylamide gel (Sigma Aldrich, Madrid, Spain) and cold electrophoresis was run. This was followed by two 30 min washes with 50 mM Tris-HCl (pH 7.4) and 2.5% Triton X-100, as well as two 10 min washes with 50 mM Tris-HCl (pH 7.4). The gel was incubated overnight at 37 °C with a buffer containing 50 mM Tris-HCl (pH 7.4), 10 mM CaCl_2_, 0.15 M NaCl, 0.1% Triton X-100, and 0.02% sodium azide. After incubation, the gels were stained for at least 1 hour with 50% methanol, 10% acetic acid, and 0.25% Coomassie blue R-250 0.25%. Finally, they were discoloured with a deinking solution containing 20% methanol and 7.5% acetic acid, at which time the white bands originating from the gelatinases were visualised on a blue background. The gels were photographed with the ChemiDoc MP Imaging System and analysed with Image Lab software (Bio-Rad, Alcobendas, Madrid, Spain). The results are shown as the percentage of optical intensity with respect to the control.

### 2.9. Viability Assays

Cell viability was assessed using the 3-(4,5-dimethylthiazol-2-yl)-2,5-diphenyltetrazolium bromide (MTT) assay. To begin with, 2 × 10^5^ cells/mL were seeded in a 96-well plate with a final volume of 100 µL and incubated for a period of 24 h. After removing the medium and replacing it with an exosome-free medium, the treatments were administered and incubated again for 24 h. Next, 6 µL of an MTT solution (1 mg/100 mL PBS) was added to the culture medium and incubated for 1.5 h in the CO_2_ incubator at 37 °C. Treatments were performed in triplicate. Subsequently, the supernatant was removed and 50 µL of dimethyl sulfoxide (DMSO) was added to dissolve the formazan crystals. Optical density was determined by measuring absorbance at 570 nm using a microplate photometer (Multiskan FC Thermo ScientificTM). Statistical analysis and IC50 values were calculated using GraphPad Prism software.

### 2.10. Migration Assays

Cells were seeded in 24-well plates at 200,000 cells/mL with a final volume of 500 µL and incubated for a period of 48 h. The wound-healing assay was performed. For this purpose, the cell monolayer was scraped off with 200 μL pipette tips and the medium was replaced with RPMI medium without exosomes. Cells were then treated with exosomes in different amounts. The wounds were photographed at regular intervals (0, 8, and 24 h), and the area of cell-free wounds was measured using ImageJ 1.38e software. All assays were carried out in triplicate.

### 2.11. Statistical Analysis

GraphPad Prism 8 software (GraphPad Software Inc., San Diego, CA, USA) was used for statistical analysis. A *t*-test was then used for comparisons between two groups, while a one-way analysis of variance (ANOVA) with Bonferroni post hoc test was used for multiple comparisons between multiple groups. The data are shown as the mean ± S.E.M. (standard error of the mean). A *p*-value of less than 0.05 was considered statistically significant.

## 3. Results

### 3.1. Biochemical Identification of Prostate Cells-Derived Exosomes

**Figure 1:**
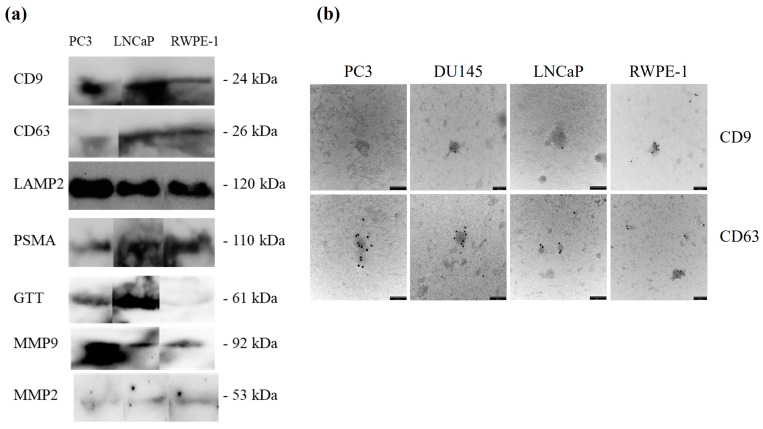
Biochemical identification of prostate cell lines-derived exosomes. (**a**) Immunodetection of CD9, CD63, LAMP2, PSMA, GGT, MMP9, and MMP2 in exosomes isolated from PC3, LNCaP, and RWPE-1 cell lines. Representative experiments are shown. (**b**) Transmission electron microscopy (TEM) of exosomes isolated from PC3, DU145, LNCaP, and RWPE-1 cell lines labelled with CD9 (top microphotographs) or CD63 (lower microphotographs). Immuno-gold labelled exosome with uranyl acetate staining. Scale bar = 100 nm.

The presence of exosomes after isolation was confirmed by immunodetection of CD9, CD63, and LAMP-2 in the prostate cell lines RWPE-1, LNCaP, and PC3. Both tetraspanins and lysosomal-associated membrane proteins were present in all exosomal fractions (Figure 1a). Subsequently, the samples were analysed by TEM. Small black dots could be identified in the images, which represent colloidal gold particles that bound to CD9- and CD63-specific antibodies present on the exosomes. These black dots indicated the presence of CD9 and CD63 proteins on the surface of the exosomes. In addition, TEM images also showed the spherical morphology of the exosomes and their structure with sizes matching those described above. TEM observation revealed prostatic vesicles with diameters ranging from 120 to 140 nm and 40 to 60 nm, confirming the presence of exosomes being marked with CD9 (Figure 1b, upper) and CD63 (Figure 1b, lower).

### 3.2. Physical Identification of Prostate Cells-Derived Exosomes

**Figure 2:**
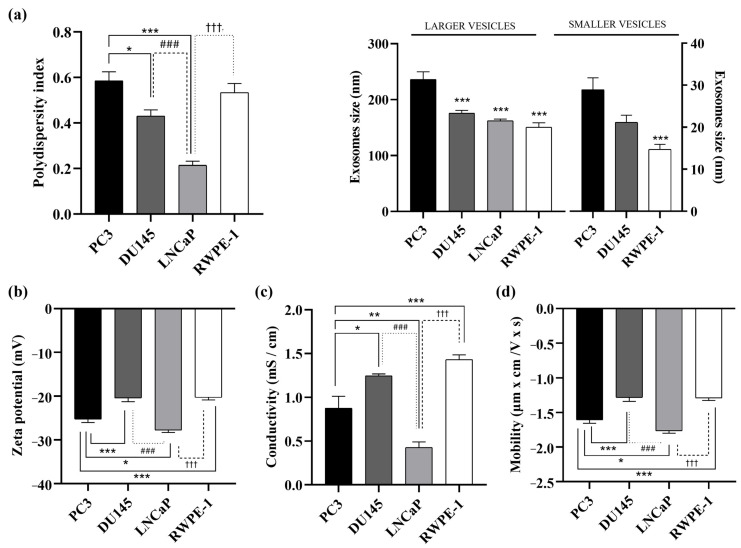
Polydispersity index, size and electrical properties of PC3-, DU145-, LNCaP- and RWPE-1-derived exosomes. (**a**) Exosomes polydispersity index and exosomes/microexosomes size (nm) isolated from culture medium of PC3, DU145, LNCaP and RWPE-1 cell lines. (**b**) Zeta potential (mV), (**c**) conductivity (mS/cm), and (**d**) mobility (µm × cm/V × s) of exosomes isolated from culture medium of PC3, DU145, LNCaP and RWPE-1 cell lines. Data represent mean ± SEM. *, *p* < 0.05; **, *p* < 0.01; ***, *p* < 0.001 compared to PC3. ###, *p* < 0.001 compared to DU145. †††, *p* < 0.001 compared to LNCaP.

DLS results showed that PC3 exosomes had a relatively large polydispersity index (PDI) of 0.59 ± 0.04 compared to DU145 and LNCaP with 0.43 ± 0.03 and 0.21 ± 0.02, respectively. No significant differences with the polydispersity index obtained with vesicles from RWPE-1 cells (0.533 ± 0.04) (Figure 2a, left panel). Two vesicle populations were obtained in PC3, DU145, and RWPE-1 cells. In a population of larger vesicles (>150 nm), exosomes derived from PC3 cells (236.1 ± 14 nm) were significantly larger in size than those obtained from vesicles derived from DU145 (175.9 ± 5 nm), LNCaP (162.3 ± 3 nm) and RWPE-1 (150.7 ± 8 nm) cells (Figure 2a, right panel). In a population of smaller vesicles (<30 nm), PC3 (28.9 ± 3 nm) cells showed larger microvesicles than those obtained from DU145 (21.1 ± 2 nm) and RWPE-1 (14.7 ± 1 nm) cells; this size was significantly larger when compared to vesicles from non-tumour cells (Figure 2a, right panel). Notably, the smaller exosome population was not detected in LNCaP cells. In Figure 2b, the zeta potential of exosomes from PC3 (−25.25 ± 1 mV), DU145 (−20.42 ± 0.5 mV), and RWPE-1 (−20.27 ± 0.5 mV) cell lines was significantly lower than the zeta potential of exosomes obtained from LNCaP (−27.75 ± 0.5 mV). In relation to the electrical conductivity derived from the resulting movement of electrically charged particles when an electric field is applied to them, we can observe that there are significant differences between all the exosomes derived from the cell lines studied (Figure 2c). Likewise, the electrophoretic mobility study indicates that the extracellular vesicles derived from all cell lines have a negative surface charge and that there are significant differences between them (Figure 2d).

### 3.3. Raman-SERS Analysis of Exosomes Isolated from Prostate Cells-Derived Culture Media

Surface-enhanced Raman spectroscopy (SERS) was performed on Whatman paper on a silver nanoparticle colloid droplet, with the aim of achieving signal amplification of the samples. The generated AgNPs were analysed by dynamic light scattering (DLS) and transmission electron microscopy (TEM) techniques to measure the size of AgNPs.

Exosome samples from prostate cancer cell lines were analysed by Raman-SERS and a subsequent chemometric analysis, to separate the samples according to their major components. Multivariate analysis by PCA allows a dataset to be analysed and classified into different groups.

**Figure 3:**
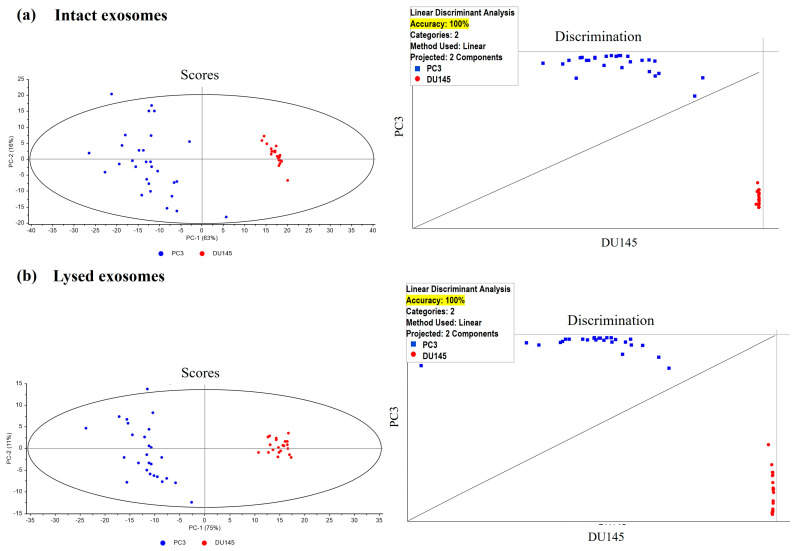
Multivariate analysis by PCA (**left** panels) and cluster analysis by PCA-LDA (**right** panels) of intact (**a**) and lysed (**b**) exosomes from castration-resistant PCa cells with moderate (DU145) or high (PC3) metastatic capacity. In both intact and lysed exosomes, the percentage accuracy according to PCA-LDA was 100%. Fifty samples were analysed: 21 from DU145 and 29 from PC3. PCA-LDA: Principal Component Analysis coupled with Linear Discrimination Analysis.

**Figure 4:**
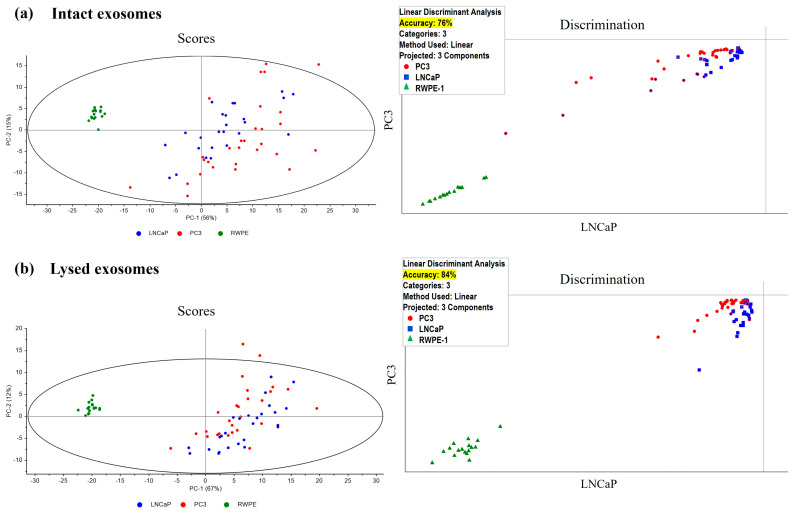
Multivariate analysis by PCA (**left** panels) and cluster analysis by PCA-LDA (**right** panels) of intact (**a**) and lysed (**b**) exosomes from RWPE (cells of human prostate epithelial), LNCaP (cells representative of castration sensible PCa) and PC3 (cells representative of metastatic castration-resistant PCa) cell lines. In intact and lysed exosomes, accuracy percentage according to PCA-LDA was 76% and 84%, respectively. A total of 72 samples were analysed: 29 from PC3, 26 from LNCaP and 17 from RWPE. PCA-LDA: Principal Component Analysis coupled with Linear Discrimination Analysis.

The analysis shows very good differentiation by components when comparing both intact or lysed exosome samples: (i) from PC3 cells versus DU145 cells (Figure 3a,b, left panels), and (ii) from RWPE versus LNCaP or PC3 (Figure 4a,b, left panels); however, there is no good differentiation by components between the exosomes of LNCaP and PC3 cells.

Because PCA analysis is an unsupervised method (i.e., no prior information is available on how to classify the sample groups), we proceeded to cluster analysis by PCA-LDA to discriminate and classify unknown samples and to ascertain the quality of the separation. The analysis of intact or lysed exosome samples from PC3 cells versus DU145 cells was performed and, in both cases, an accuracy of 100% was obtained (Figure 3a,b, right panels). In addition, PCA-LDA analysis of RWPE cells versus LNCaP cells versus PC3 cells showed an accuracy of 76% and 84%, respectively, for intact and lysate exosome samples (Figure 4a,b, right panels).

### 3.4. Activity of Gamma-Glutamyl Transpeptidase in Prostate Cells-Derived Exosomes

**Figure 5:**
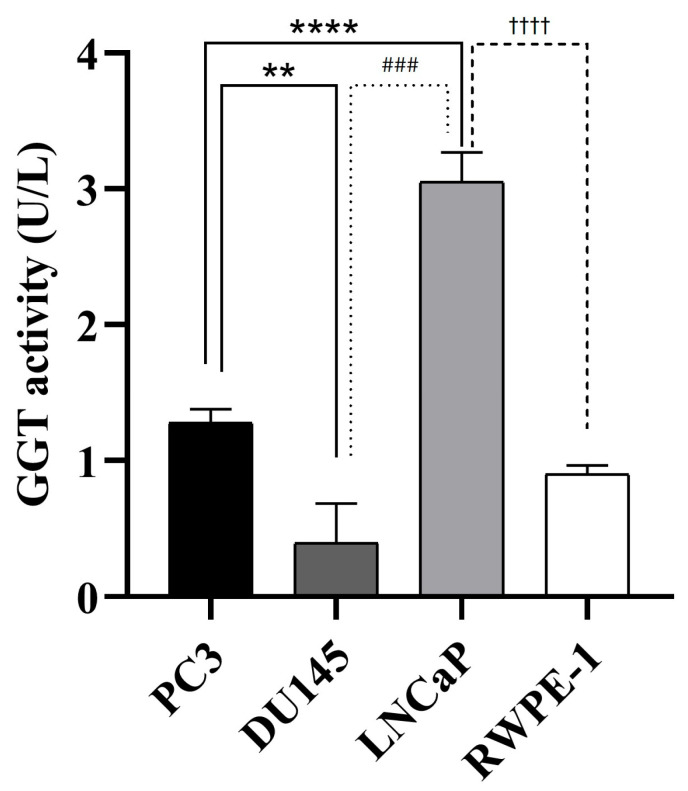
Gamma-glutamyl transpeptidase (GGT) activity in exosomes isolated from culture medium of PC3, DU145, LNCaP and RWPE-1 cell lines. A highly significant almost 3-fold increase in activity of exosomes isolated from the LNCaP cell line versus PC3 is shown. Data represent mean ± SEM. **, *p* < 0.01; ****, *p* < 0.0001 compared to PC3. ###, *p* < 0.001 compared to DU145. ††††, *p* < 0.0001 compared to LNCaP.

In addition, the GGT activity of exosomes isolated from prostate cell lines was measured. A significant increase in GGT activity was observed in LNCaP cells (3.05 ± 0.2 U/L) compared to PC3 (1.27 ± 0.1 U/L), DU145 (0.39 ± 0.3 U/L), and RWPE-1 (0.91 ± 0.06 U/L) cells (Figure 5).

### 3.5. Gelatinase Activity Measurement of Prostate Cells-Derived Exosomes

**Figure 6:**
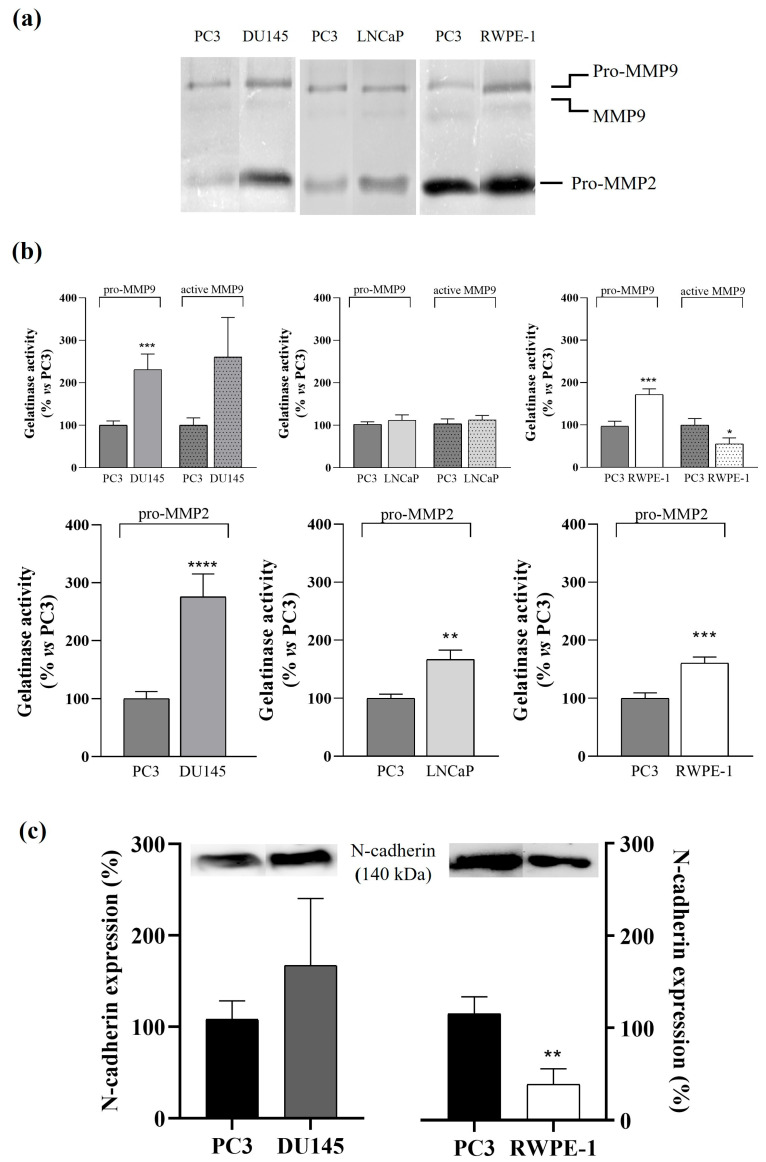
Gelatine zymography of matrix metalloproteinases 9 and 2 from prostate cell lines-derived exosomes. (**a**) Zymographs were performed in PC3 and DU145, LNCaP, or RWPE-1 cell lines. Very slight bands of MMP9 (84 kDa) and high levels of pro-MMP9 (92 kDa) and pro-MMP2 (74 kDa) were detected in all cell lines. Each zymogram is representative of six independent experiments. (**b**) Densitometric analysis of zymography gels from six separate experiments, showing the percentage of gelatinase activity of isolated exosomes from DU145, LNCaP, or RWPE-1 cell lines with respect to PC3 gelatinase activity. Data represent mean ± SEM. *, *p* < 0.05; **, *p* < 0.01; ***, *p* < 0.001; ****, *p* < 0.0001. (**c**) N-cadherin expression in exosomes isolated from culture medium of PC3, DU145 and RWPE-1 cell lines. Data represent mean ± SEM of N-cadherin expression percentage with respect to PC3. **, *p* < 0.01. Representative experiments are shown.

The proteolytic activity of the metalloproteases MMP2 and MMP9 was assessed by zymography using exosomal extracts from PC3, DU145, LNCaP, and RWPE-1 cells. This technique allowed us to observe the presence of both the active form and the pro-form of MMP9, revealing two bands of approximately 82 and 92 kDa, respectively, and a 72 kDa band corresponding to pro-MMP2 (Figure 6a). However, no active MMP2 was identified in the samples analysed. The isoforms detected by zymography were assessed by band densitometry comparing PC3 cell lines with DU145, LNCaP, and RWPE-1 cells (Figure 6b). In DU145 cells, the gelatinase activity of both pro-isoforms was significantly increased (2.31-fold for pro-MMP9 and 2.75-fold for pro-MMP2) compared to those obtained in PC3 cells. In LNCaP cells, only the activity of pro-MMP2 isoform was significantly increased (1.7-fold) compared to that obtained in PC3. In RWPE-1 cells, the gelatinase activity of both pro-isoforms was significantly increased (1.8-fold for pro-MMP9 and 1.7-fold for pro-MMP2) compared to those obtained in PC3 cells. The activity of the active MMP9 isoform was significantly decreased compared to the PC3 cells.

### 3.6. Effect of Treatment with PC3-, LNCaP-, or RWPE-1-Derived Exosomes on PC3 Cell Viability

**Figure 7:**
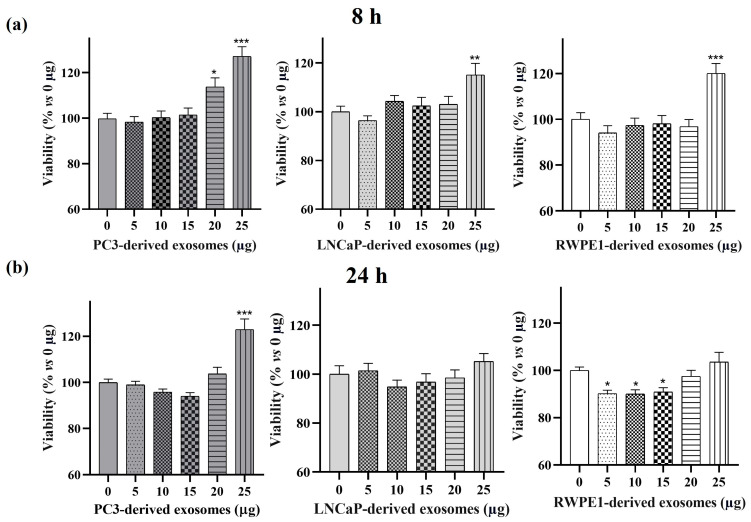
PC3 cells viability treated with exosomes isolated from culture medium of prostate cell lines. PC3 cells were treated for 8 h (**a**) or 24 h (**b**) with 0 µg, 5 µg, 10 µg, 15 µg, 20 µg, or 25 µg of RWPE1-, LNCaP, or PC3-derived exosomes. Data represent mean ± SEM of cell viability percentage with respect to 0 μg exosomes. *, *p* < 0.05; **, *p* < 0.01; ***, *p* < 0.001 compared to 0 µg exosomes.

To evaluate the effect of exosomes from cells representative of different stages of prostate cancer (PC3, LNCaP, and RWPE-1), cell viability studies were performed on the PC3 line. As shown in Figure 7, at 8 h of treatment, a significant (1.13–1.3-fold) increase in PC3 cell viability was observed after exosome treatment of the three cell lines studied (25 μg for LNCaP and RWPE-1; 20 and 25 μg for PC3) compared to the control. At 24 h, RWPE-1 exosome treatments (5–15 μg) showed a 10% inhibitory effect on PC3 cell viability compared to the control; and PC3 exosome treatment significantly increased PC3 cell viability (1.3-fold).

### 3.7. Effect of PC3-Derived Exosomes Treatment on PC3 Cell Migration

**Figure 8:**
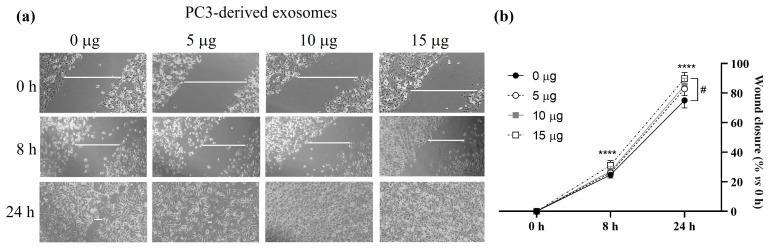
Effect of PC3-derived exosomes on PC3 cell migration. PC3 cells were treated for 0 h, 8 h or 24 h with 0 µg, 5 µg, 10 µg, or 15 µg of PC3-derived exosomes. (**a**) Wound-healing assay was performed to detect the migration of cells. Representative images are shown from three independent experiments. (**b**) The percentage of wound closure at different times (0 h, 8 h and 24 h) compared to the initial wound (0 h) is shown. Data represent mean ± SEM. ****, *p* < 0.0001 compared to 0 h. #, *p* < 0.05 compared to 0 µg exosomes.

Cell migration is one of the most relevant processes in tumour progression. Through the wound closure assay, we investigated whether treatment with increasing amounts of PC3 cell-derived exosomes promotes the migration of this cell line. As shown in Figure 8, at the start of the experiment (0 h), the wound size was very similar in all groups. After 24 h of incubation, with and without exosomes, PC3 cells migrated and filled the wound to different degrees. Figure 8 shows the distance travelled by the cells in each case, with significantly greater (*p* < 0.001) wound closure observed at 8 and 24 h after treatment with their own exosomes compared to the control. Thus, after 8 h of treatment, cells treated with 5, 10, or 15 µg of PC3-derived exosomes showed a wound closure of 26.06%, 27.13%, or 31.14%, respectively, which is a higher percentage compared to the control, which was 24.72%. At 24 h, wound closure was 82.82%, 85.06% and 89.79% for cells treated with 5 µg, 10 µg and 15 µg of exosome, respectively, significantly higher than the 75.05% observed in untreated cells.

## 4. Discussion

Extracellular vesicles are secreted by a wide variety of cells, both tumour and non-tumour cells, that play an important role in the development and progression of cancer. They can be isolated from a variety of body fluids, making them promising tools for the diagnosis and treatment of a given disease. Different subtypes of extracellular vesicles have been described according to their size: small extracellular vesicles (S-EV) or exosomes below 200 nm, and large extracellular vesicles (L-EV) or oncosomes above 200 nm. Differences in exosome membrane composition and exosome content according to origin have also been described [6,21]. In the present study, exosomes from several prostate cell lines were isolated and characterised. The lines correspond to a non-tumour control situation (RWPE-1) and to different stages of PCa: (i) hormone-refractory with high (PC3) and moderate (DU145) metastatic potential, and (ii) hormone-dependent or less aggressive (LNCaP). First, the exosomes obtained by ultrafiltration-filtration were characterised by various techniques, such as Western blotting, TEM, DLS, and ELS. Validation of exosome isolation was performed by immunodetection of two characteristic exosome markers (CD9 and CD63) and the glycoprotein LAMP2, ensuring the reliability of the isolation method used [22]. TEM using antibodies against CD9 and CD63 revealed the presence of S-EV in all lines studied. In this regard, larger L-Evs have been reported to contain more DNA, CD9, or Annexin A1, while S-Ves or exosomes contain CD63, CD9, and CD81 [18]. DLS showed heterogeneity in the size of exosomes in the cell lines. Thus, the PC3, DU145, and RWPE-1 lines had populations of two different sizes (150–235 nm and 15–30 nm). In both cases, highly metastatic androgen-independent prostate cancer cells (PC3) had the largest exosome size and non-tumourigenic prostate cells (RWPE-1) had the smallest exosome size. These results are in line with other studies carried out in pancreatic, breast, and brain cancer, which show the existence of two populations of vesicles, smaller and larger in diameter, the latter being related to cancerous processes [23,24]. In relation to prostate cancer lines, one type of population has been described in PC3, DU145, and LNCaP with a diameter of 100 nm [25]. Meanwhile, other groups have detected more population types in PC3 cells within a size range of 30–200 nm [26]. Of note, androgen-dependent prostate cancer cells (LNCaP) had only one vesicle population (162 nm). In this regard, we characterised serum exosomes from patients with and without prostate cancer in previous studies. These also showed two populations of exosomes with a hydrodynamic radius of 140–170 nm and 20–25 nm [27]. The population of exosomes with a size of 15–30 nm in diameter, obtained in our study, may be microexosomes, described as structures with the same components as exosomes but whose formation process and sizes are different [28,29]. Furthermore, exosomes have been given a pathological role and microexosomes a physiological role.

The membrane of EVs is a highly interactive and dynamic surface that interacts with the extracellular environment [30]. The interactions of small eVs act as signalling modulators in biological processes, such as metastasis and cancer regulation [31]. The introduction of zeta potential measurement in the direct characterisation of extracellular vesicles has not been widely used to date. However, the need to provide a translational perspective and improve the characterisation of exosomes in the clinical setting has been reported in some publications [32,33]. In this regard, we incorporated such measurements in the characterisation of exosomes from cell lines representing different stages of prostate cancer (PC3, DU145 and LNCaP) and from a control situation (RWPE-1). We found significant differences in the charge of these exosomes in prostate cancer cells compared to control cells. In this sense, the exosomes of the less aggressive stages correspond to more stable and less aggregated vesicles, which would facilitate transport and dissemination to other sites. Subsequently, the experimentally determined zeta potential was used to estimate both the electrophoretic mobility and the membrane conductance of the exosomes. The study of the electrophoretic mobility of the exosomes evaluated showed significant differences in the cells that represent the progression of prostate cancer. This would be a key factor in the approach to establishing the diagnosis and prognosis of the disease. In addition, the different physical properties of extracellular vesicles would be very useful in the development of standardised protocols for the characterisation of exosomes from clinically relevant sources (human plasma, serum, or urine) [34]. When the conductivity of the exosomes of the cells studied was estimated, it was revealed that they had different conductivity. Thus, exosomes derived from control cells had a higher conductivity than exosomes derived from prostate cancer cells, giving these vesicles an important role as diagnostic biomarkers of prostate cancer. In addition, there were also significant differences in the measurement of conductivity between tumour cell exosomes, which could be very useful in establishing disease prognosis. Likewise, these results could determine the use of techniques such as dielectrophoresis to separate and identify exosomes in samples with clinical relevance [35].

The differences obtained in the surface charge of the exosomes studied and consequently in their composition are confirmed by the results obtained in Raman-SERS studies of the extracellular vesicles. Our work demonstrates that the PCA-LDA-based Raman-SERS technique can be used for the direct detection and identification of exosomes derived from cultured cells with excellent specificity and sensitivity. A diagnostic sensitivity of 76% can be achieved for the differentiation of three types of prostate exosomes. In turn, analysis of non-lysed exosome components derived from cell lines representing castration-resistant stages shows that there are differences in the composition of highly metastatic (PC3) and low metastatic potential (DU145) pCa cells, with 100% accuracy. This could involve a significant difference in metabolites or small molecules (such as miRNA or mRNA) or in the amount of them between the two cell lines. Similar results were obtained when studying lysed exosomes (Figure 3b and Figure 4b). Raman-SERS spectroscopy can be used as an ultra-sensitive (single-cell level), non-invasive, and rapid method that provides valuable structural and biochemical analysis of extracellular vesicles [36]. In addition, Raman spectroscopy coupled with multivariate techniques provides the statistical diagnostic and prognostic approach for efficient screening of exosomes.

Gamma-glutamyl transferase (GGT) contributes to the metabolism of glutathione (GSH), an important intracellular antioxidant, by cleaving its glutamyl bond and releasing cysteinyl-glycine and a γ-glutamyl amino acid. Overexpression of GGT promotes proliferation in cancer cells by recycling cysteine from extracellular GSH, thereby increasing resistance to oxidative stress [21]. Furthermore, GGT expression has been reported to be associated with drug resistance, possibly because several drugs bind GSH, the presence of which is increased by GGT activity. Recent studies in the field of epidemiology have indicated that GGT activity may serve as an indication of increased chances of developing prostate cancer [37]. GGT expression and activity have also been shown to be increased in exosomes isolated from PCa cell lines and exosomes derived from the serum of PCa patients [38]. Our findings, based on photometry of exosome samples from all cell lines studied, support the presence of GGT activity. The enzyme activity values obtained in the hormone-dependent cell line (LNCaP) were higher than those of the hormone-independent lines (PC3 and DU145). This could support the fact that in the early stages of prostate cancer GGT activity is required to favour disease progression. It is also important to note the results obtained by Kawakami et al. [38] who showed a significant increase in serum exosomal GGT activity among patients with PCa compared to those with benign prostatic hyperplasia (BPH). These findings indicate that serum exosomal GGT activity could potentially serve as a biomarker to differentiate between patients with PCa and patients with BPH, whose PSA levels are similar. In previous studies, we described that serum exosomes from PCa patients with Gleason grades 6 and 7 increased GGT activity in LNCaP and PC3 cells [27].

Epithelial-mesenchymal transition (EMT) is a process that allows epithelial cells to acquire mesenchymal properties, like fibroblasts, with reduced intercellular adhesion and increased motility. The invasive and metastatic behaviour of epithelial cancer cells depends on the acquisition of EMT features such as overexpression of extracellular matrix (ECM) proteolytic enzymes and increased N-cadherin, which contributes to increased tumour cell motility and invasive properties. The activity of MMP9 and/or MMP2 proenzymes was increased in all representative stages of prostate cancer progression. We have described in previous studies that after incubation of LNCaP and PC3 cells with serum exosomes from PCa patients with Gleason grade ≥7, an increase in the activity of MMP9 and MMP2 proenzymes was observed [27]. In relation to N-cadherin, a non-significant increase was observed in androgen-independent prostate cancer cells (Figure 6c).

Regarding cell viability, the treatment of PC3 cells for 8 h with 25 µg of the exosomes isolated from the three prostate cell lines significantly increased cell viability. This effect was not maintained at 24 h of treatment, as incubation with exosomes isolated from RWPE-1 and LNCaP showed no significant difference in cell viability compared to the control. However, treatment of PC3 cells with 5, 10, or 15 µg protein of RWPE-1 derived exosomes significantly decreased cell viability at 24 h. The differences in the observed results could be attributed to the molecular heterogeneity of the exosomes of the different cell lines. This heterogeneity may lead to different effects on target cells due to the different expression of cell surface receptors on each type of exosome [39].

On the other hand, treatment of PC3 cells with PC3-exosomes induced an increase in cell migration, a crucial aspect in tumour progression. This was evidenced by the wound closure assay, where it was observed that treatment of the prostate cancer cell line with the exosomes promoted faster wound closure compared to the control group, being significant at 15 µg of exosomes. This finding is consistent with the existing literature, which suggests that exosomes can facilitate cell migration and invasion, two key processes in cancer metastasis [40]. In studies conducted by our group, serum exosomes from PCa patients significantly enhanced wound healing at 8 and 24 h, whereas the response induced by treatment with exosomes from non-PCa patients was virtually identical to the response of control cells [27]. The ability of exosomes to influence these processes highlights their potential as mediators of PCa progression and as potential therapeutic targets.

## 5. Conclusions

Exosomes may be potential biomarkers for the diagnosis and prognosis of PCa through their biochemical and biophysical characterisation. The activity of exosome enzymes such as gamma-glutamyl transferase (GGT) and metalloproteinases (MMPs) may be associated with tumour aggressiveness and poor prognosis. The analysis of exosomes isolated from biological samples (p.e. urine or serum) of PCa patients by Raman-SERS spectroscopy, as well as the characterisations of their biophysical properties, seem to be promising tools to allow a non-invasive assessment of the disease. In summary, exosomes represent a promising field of research in the diagnosis, prognosis, and treatment of prostate cancer.

## Figures and Tables

**Table 1:** Primary and secondary antibodies used.

Primary Antibody	Species	Dilution	Supplier
anti-CD9	mouse	1:200	Santa Cruz Biotechnology (Heidelberg, Germany)
anti-CD63	mouse	1:200	Santa Cruz Biotechnology
anti-LAMP2	mouse	1:200	Santa Cruz Biotechnology
anti-GGT	rabbit	1:1000	Abcam (Cambridge, UK)
anti-PSMA	rabbit	1:5000	Abcam
anti-MMP9	mouse	1:200	Santa Cruz Biotechnology
anti-MMP2	mouse	1:200	Santa Cruz Biotechnology
anti-N-cadherin	rabbit	1:500	Invitrogen (Alcobendas, Madrid, Spain)

## Data Availability

All figures and data are included in the manuscript.
